# Editorial: How pharmacogenomics, epigenetics, and data analysis could improve anticancer treatment?

**DOI:** 10.3389/fphar.2022.1067022

**Published:** 2022-11-10

**Authors:** Abdeslam Jaafari, Subramani Srinivasan, Mounir Tilaoui

**Affiliations:** ^1^ Laboratory of Biological Engeneering, Sultan Moulay Slimane University, Beni Mellal, Morocco; ^2^ Lab of Bioprocess-Biointerfaces, Sultan Moulay Slimane University, Beni Mellal, Morocco; ^3^ Department of Biochemistry and Biotechnology, Faculty of Science, Annamalai University, Chidambaram, Tamilnadu, India; ^4^ Postgraduate and Research Department of Biochemistry, Government Arts College for Women, Krishnagiri, Tamilnadu, India; ^5^ Pharmaceutical and Molecular Biotechnology Research Center, Waterford Institute of Technology, Waterford, Ireland

**Keywords:** cancer, pharmacogenomics, data analysis, epigenetics, bioinformatics

We need more than classical weapons to fight a dreadful disease like cancer. Other emergent sciences like epigenetics, pharmacogenomics, or data analysis and bioinformatics could play a vital role in this sense. The goal of our research topic is to give more insights into the relationship between pharmacogenes and drug response, new genes involved in anticancer drug effects and/or side effects, how drug repurposing could help in the study of the interaction between the genome and anticancer drug response, how could epigenetic modification be involved in the interaction between pharmacogenes and anticancer drug effects, and how could data analysis support in understanding and predicting the relationship between pharmacogenes and drug response.

As there is no doubt today that epigenetic modifications are involved in cancer pathogenesis, progress, and prognosis, researchers are looking for ways to treat cancer by fixing these epigenetic alterations (F.H. Sarkar, 2013, Kanwal and Gupta, 2010, Soo You and Jones, 2012). In fact, this approach could provide good results since epigenetic modifications, unlike genetic mutations, are reversible. An original article on our topic entitled *Decitabine-induced DNA methylation-mediated transcriptomic reprogramming in human breast cancer cell lines*; *the impact of DCK overexpression* reported that decitabine (DNA methyltransferase (DNMT) inhibitor) induced hypermethylation and down-regulation of some genes in two breast cancer cell lines. The authors concluded that decitabine has broad reprogramming abilities that could normalize the aberrant transcriptional profiles in cancer cells (Buocikova et al.). In another study entitled *Establishment*, *immunological analysis*, *and drug prediction of a prognostic signature of ovarian cancer related to histone acetylation*, it has been reported that histone acetylation modulators, such as HDAC1, HDAC10, and KAT7, can act as independent prognostic factors for ovarian cancer and are related to poor prognosis (Fang et al.).

On the other hand, it has been confirmed that the patient’s genotype could highly impact the therapeutic effect and/or adverse events of anticancer drugs in particular (Filipski et al., 2014; Hlavác et al., 2020). Pharmacogenomics/pharmacogenetics aims to evaluate the relationship between drug efficacy/toxicity of a given drug and its pharmacokinetics and pharmacodynamics. Proteins involved in all these mechanisms are encoded by genes called pharmacogenes. Thus, any mutation in those genes could lead to treatment failure and/or resistance of cancer cells to chemotherapeutic drugs. Consequently, we can optimize and improve anticancer drug efficacy and/or adverse effects by understanding the interaction between genome variation and drug response (Hlavác et al., 2020; Kelly et al., 2014; Wheeler et al., 2012). Recently, the next-generation sequencing technology has led to the discovery of new genetic variants, such as cytochrome P450 genes and ATP-binding cassette (ABC) transporters, related to anticancer therapy and cancer cell resistance, the major obstacle to successful anticancer treatment (Wheeler et al., 2012). In the same study by Fang et al., it has been reported that the response to anticancer immunotherapy could be influenced by the patient’s genotype. In fact, patients in the high-risk group had a higher likelihood of immune escape or rejection and were less likely to respond to platinum/paclitaxel therapy (Fang et al.).

Bioinformatics and data analysis could also help in providing some vital information, which can lead to elucidating the genome–drug response relationship to find the right drug for the right patient in the treatment and prevention of cancer (Dezso and CeccareOlsen et al., 2020; Olsen et al., 2014). In their study *Network pharmacology-based investigation and experimental validation of the mechanism of scutellarin in the treatment of acute myeloid leukemia*, Huang et al. used some public databases, such as PharmMapper, UniProt, OMIM, GeneCards, DrugBank, and PharmGKB databases, to determine the potential targets of scutellarin in AML (acute myeloid leukemia). On the other hand, protein–protein interaction (PPI), Gene Ontology (GO), and Kyoto Encyclopedia of Genes and Genomes (KEGG) pathway enrichment analysis were conducted to uncover the mechanism of scutellarin in the treatment of AML. By integrating network pharmacology-based prediction and experimental validation, the authors, in this study, concluded that the JNK pathway plays a crucial role in scutellarin-mediated AML treatment (Huang et al.).

In another research work on this topic entitled *Examination on the risk factors of cholangiocarcinoma: A Mendelian randomization study*, the authors used Mendelian randomization to study the involvement of 26 risk factors in CCA (cholangiocarcinoma). For each factor, genetic variants were obtained from their respective GWAS. The estimation of this relationship was performed using the inverse variance-weighted (IVW) average method (Chen et al.).

Fang et al., in their study entitled *Establishment*, *immunological analysis*, *and drug prediction of a prognostic signature of ovarian cancer related to histone acetylation*, applied LASSO regression and the Cox algorithm to determine a prognostic signature for ovarian cancer associated with histone acetylation modulator genes. Moreover, they performed an immunological bioinformatics analysis of the model from multiple perspectives using the CIBERSORT algorithm, ESTIMATE algorithm, and TIDE algorithm to verify the model’s accuracy (Fang et al.).
